# ﻿A fusarioid fungus forms mutualistic interactions with poplar trees that resemble ectomycorrhizal symbiosis

**DOI:** 10.3897/imafungus.16.143240

**Published:** 2025-03-07

**Authors:** Ningning Yang, Xiaoliang Shan, Kexuan Wang, Junkun Lu, Ying Zhu, Redman S. Regina, Russell J. Rodriguez, Jiajia Yao, Francis M. Martin, Zhilin Yuan

**Affiliations:** 1 State Key Laboratory of Tree Genetics and Breeding, Chinese Academy of Forestry, Beijing 100091, China Research Institute of Subtropical Forestry, Chinese Academy of Forestry Hangzhou China; 2 College of Forestry, Nanjing Forestry University, Nanjing 210037, China State Key Laboratory of Tree Genetics and Breeding, Chinese Academy of Forestry Beijing China; 3 Zhejiang Key Laboratory of Forest Genetics and Breeding, Hangzhou 311400, China Zhejiang Key Laboratory of Forest Genetics and Breeding Hangzhou China; 4 Research Institute of Subtropical Forestry, Chinese Academy of Forestry, Hangzhou 311400, China Nanjing Forestry University Nanjing China; 5 College of Plant Protection, Nanjing Agricultural University, Nanjing 21004, China Nanjing Agricultural University Nanjing China; 6 Research Institute of Tropical Forestry, Chinese Academy of Forestry, Guangzhou 510520, China Research Institute of Tropical Forestry, Chinese Academy of Forestry Guangzhou China; 7 Institute of Biology, Gansu Academy of Sciences, Lanzhou 730000, China Institute of Biology, Gansu Academy of Sciences Lanzhou China; 8 Adaptive Symbiotic Technologies, University of Washington, Seattle, WA 98195, USA University of Washington Seattle United States of America; 9 INRA, UMR 1136 INRA-Université de Lorraine ‘Interactions Arbres/Microorganismes’, Laboratoire d’Excellence ARBRE, Centre INRA-Lorraine, Champenoux, France INRA-Université de Lorraine ‘Interactions Arbres/Microorganismes’ Champenoux France

**Keywords:** Convergent evolution, endophytic fungi, lifestyle switch, root-fungal symbioses, synthetic mutualism

## Abstract

*Fusarium* species, recognised as global priority pathogens, frequently induce severe diseases in crops; however, certain species exhibit alternative symbiotic lifestyles and are either non-pathogenic or endophytic. In this study, we characterised the mutualistic relationship between the eFp isolate of *F.pseudograminearum* and five poplar species, resulting in formation root structures reminiscent of ectomycorrhizal (ECM) symbiosis. This functional symbiosis is evidenced by enhanced plant growth, reciprocal nutrient exchange, improved nitrogen and phosphorus uptake and upregulation of root sugar transporter gene expression (*PtSweet1*). Comparative and population genomics confirmed that eFp maintains a structurally similar genome, but exhibits significant divergence from ten conspecific pathogenic isolates. Notably, eFp enhanced the growth of diverse plant lineages (*Oryza*, *Arabidopsis*, *Pinus* and non-vascular liverworts), indicating a near-complete loss of virulence. Although this specialised symbiosis has only been established *in vitro*, it holds significant value in elucidating the evolutionary track from endophytic to mycorrhizal associations.

## ﻿Introduction

Mycorrhizal fungi, the primary components of plant root mycobiomes, have co-evolved with plants for a minimum of 400 Mya and are essential for host nutrient uptake and enhanced fitness under adverse environmental conditions (Martin et al. 2017). An extension of this mutualism has been observed in plant–endophytic fungal associations (Rodriguez et al. 2009), which are particularly significant for non-mycorrhizal plants or plants inhabiting extreme environments (Almario et al. 2017, 2022; Yuan et al. 2021). Analogous to what we observed in other types of mutualism, genotype-by-genotype by-environment (G × G × E) interactions, within the framework of geographic mosaic theory of co-evolution (Thompson 2019), could extensively impact the degree of root–fungus mutualism stability and effectiveness. Consequently, the outcome of these associations can fluctuate along the mutualism–parasitism continuum.

Several empirical studies have emphasised that individuals within mycorrhizal fungal populations exhibit substantial heritable genetic variations, contributing to physiological changes and indirectly influencing symbiotic traits. For instance, intraspecific genetic polymorphisms, specifically copy number variations (CNVs), have been shown to affect host growth in the arbuscular mycorrhizal fungus *Rhizophagusirregularis* (Wyss et al. 2016). Notably, the implications of within-fungus variation are complex in certain root-endophytic guilds. Accumulating evidence suggests that strains of endophytic fungi isolated from healthy plants are often genetically similar to their well-known pathogenic relatives (Fesel and Zuccaro 2016; Hacquard et al. 2016). Most endophytes belonging to the *Fusarium* and *Colletotrichum* genera are frequently observed in plants and experimental evidence has confirmed their positive effects on plant fitness, indicating symbiotic lifestyle switching (Redman et al. 2001; Hiruma et al. 2016; Lofgren et al. 2018). Transcriptomic signatures reveal the downregulation of pathogenicity-related genes or disruption of toxin biosynthesis (Hacquard et al. 2016). However, traits related to saprotrophic or pathogenic lifestyles may still be retained in the genome as they are potentially detrimental when transitioning to new host plants. This notion is supported by several recent studies (Hernandez-Escribano et al. 2018; Nelson et al. 2018; Tian et al. 2020).

Relationships spanning parasitic–endophytic interactions appear to be common and such endophytes have not yet diverged sufficiently from their pathogenic relatives (Hill et al. 2022). However, we observed that two endophytic *Fusarium* species, *F.culmorum* and *F.pseudograminearum* (eFp), isolated from the dunegrass *Leymusmollis* in a high-salinity beach habitat, exhibited plant growth-promoting traits and enhanced salt tolerance (Rodriguez et al. 2008; Redman et al. 2011). Notably, during the co-cultivation of *F.pseudograminearum* and poplar trees under gnotobiotic conditions, this fungus initiated the development of root structures resembling those developed during ectomycorrhizal (ECM) symbiosis and significantly promoted plant growth.

Thus, it appears that eFp acts as a specialised mutualist and we investigated the morphological and physiological attributes associated with this association and compared the similarities and differences with genuine ECM symbioses. We posited that *F.pseudograminearum* might interact with poplar trees in an isolate-specific manner. This hypothesis was examined by integrating data from axenic synthesis trials, anatomical and isotope tracing assays and experimental inoculations. Furthermore, it was hypothesised that *F.pseudograminearum* is genetically distinct from other conspecific pathogens.

## ﻿Materials and methods

### ﻿Poplar cuttings and fungal materials

To demonstrate the degree of potential compatible interactions between *F.pseudograminearum* isolate eFp and poplar trees, five poplar clones were used in this work including *P.tomentosa*, *P.trichocarpa*, *P.alba*, *P.alba* × P.tremulavar.glandulosa 84k and *P.deltoides* × *P.euramericana* NL895. Inoculation tests were performed with clones of poplar cuttings that were micropropagated *in vitro* in the laboratory. The media composition for micropropagation of each clone is provided in Suppl. material 1: table S1. eFp was originally isolated from the healthy halophyte *Leymusmollis* (Rodriguez et al. 2008) and was routinely maintained on potato dextrose agar (PDA; BD Difco).

To further evaluate the plant growth-promoting activity of eFp in a wide range of plants, we selected the following plants for inoculation: *Arabidopsisthaliana* (Col-0 ecotype), rice (*Oryzasativa*), liverwort (*Marchantiapolymorpha*, wild-type accession Takaragaike-1), *Pinuselliottii* and *Pinusmassoniana*. The method for the propagation of *M.polymorpha* was provided by Redkar et al. (2022a) and seeds of other plants were surface-infected for germination and growth. Pathogenic *F.pseudograminearum* isolate Fp8 was used as a negative control for the plant growth assay.

### ﻿Artificial synthesis of eFp–poplar associations *in vitro*

It has been proposed that nutrient conditions can significantly influence mycorrhizal formation. Generally, high glucose, low phosphorus concentrations (50 μM KH_2_PO_4_) as well as a low concentration of 2-(N-morpholino) ethanesulphonic acid (MES) buffer (1.25 mM, pH 5.6) was applied to promote ectomycorrhization (Guerin-Laguette et al. 2000). Given the fast growth rate of eFp, the glucose concentration was kept at a 2 g l^-1^. In addition, organic nitrogen, composed of a mixture of acidic, neutral and aromatic amino acids [glutamine (Glu), glycine (Gly), valine (Val), leucine (Leu) and phenylalanine (Phe)], was added to the base medium at a final concentration of 3.57 mM N.

### ﻿Microscopy and anatomical description of the eFp-root association

To characterise the infection pattern and growth of eFp within poplar roots, we collected the well-developed eFp-colonised root tips 40 days after inoculation and observed their potential functional structures. First, the gross morphology of the ECM-like root tips was examined using a 3D ultra-depth stereoscopic microscope VHX-5000 (Keyence, Osaka, Japan). Next, a subset of 80% (v/v) ethanol-fixed root samples was sectioned. Briefly, the roots were washed three times with PBS (pH 7.4) and embedded in Tissue OCT-Freeze Medium (Sakura Finetek USA, Inc., Torrance, CA, USA). Transverse sections (8–10 µm in thickness) were prepared using a Thermo Cryostar NX70 freezing microtome (Thermo Fisher Scientific, Walldorf, Germany) and dual staining of the fungal cell wall (wheat germ agglutinin-alexa fluor, WGA Alexa-Fluor 488, Thermo Fisher Scientific, MA, USA) and plant cell wall (Propidium Iodide, PI, Sigma-Aldrich, USA) was performed (Becker et al. 2018). Serial sectioning was performed to avoid the sectioning of artefacts. The samples were immersed in staining solution (20 µg ml^-1^ PI, 10 µg ml^-1^ WGA Alexa-Fluor 488, and 0.1% Tween 20 dissolved in PBS) for 4 h. All sections were viewed at 400–1000 × magnification under a confocal laser scanning microscope (LSM 980 with Airyscan 2; Carl Zeiss, Jena, Germany) equipped with ZEN2 software.

### ﻿Measurement of poplar growth and nutrient uptake

Prior to fungal inoculation, uniform *P.tomentosa* cuttings were transferred to containers (10 cm in diameter and 18 cm in height) with nutrient agar and the poplar roots were inoculated with eFp by spraying 200 μl of 1 × 10^4^ spores/ml into the medium, while the fungus-free controls were mock-inoculated with sterile water (five replicates, five plantlets per replicate per treatment). Nutrient agar was composed of 1/10 Murashige and Skoog (MS) medium (Murashige and Skoog 1962) and 1/5 potato broth containing glucose and sucrose (2 g l^−1^). Plants were kept in a growth chamber under a 12 h light/12 h dark photoperiod at 25 °C with a light intensity of approximately 5,000 lx. Forty days post-inoculation, the plant height, stem diameter, shoot-root ratio and biomass of the treated plants were measured and compared. The total root length, root surface area, root volume, number of root tips and number of root forks of intact roots were rinsed with tap water and scanned (Epson Expression 11000XL, Epson, CA, USA) using image analysis software (WinRHIZO Pro version, Regent Instruments, Quebec, Canada). Chlorophyll content was determined as described by Peng et al. (2021). The nitrogen content of the shoot samples was determined using the Kjeldahl nitrogen determination method with sulphuric acid-hydrogen peroxide digestion (Bradstreet 1954) and the phosphorus content was determined using the molybdenum-antimony resistance colourimetric method (Tiessen and Moir 1993).

### ^﻿14^C and ^15^N isotopic tracer experiments

To further confirm the occurrence of mutualistic interactions between eFp and *P.tomentosa* cuttings, stable isotope labelling experiments using ^13^C and ^15^N were performed to trace the bidirectional transfer of C and N between the plants and the fungus (Kaiser et al. 2015). Detailed methods for establishing microcosms can be found in Thoen et al. (2010). In brief, 90-mm split Petri dishes were used to create a plant and a fungal compartment in each microcosm, with a barrier to prevent leakage of tracers between them. Nylon mesh (8.5 cm long × 0.8 cm wide) in the middle of split Petri dishes acted as a barrier to avoid the growth of plant roots into the fungal compartment. The plant compartment was filled with 25 ml of nutrient agar and the roots cuttings were restricted to the plant compartment. The shoot was outside the dish (a small hole made by a hot scalpel) and the cutting was inoculated with a mycelial plug before the ^13^C pulse, whereas a 5 mm diameter mycelial plug was only placed on the ^15^N-labelled agar in the fungal compartment. The fungal side of the ^13^C labelling microcosm was loaded with 25 ml PDA. Two-day ^13^C pulse-labelling, produced through a reaction between Ba^13^CO_3_ (98 atom% ^13^C, Macklin, Shanghai, China) and lactic acid (Wang et al. 2013), was supplied to a leaf chamber. For the ^15^N pulse-chasing experiment, a small 35 mm Petri dish, preventing leakage of the tracers to the surrounding agar, was equipped with 9.5 ml of malt extract agar (MEA) containing the ^15^(NH_4_)_2_SO_4_ (99 atom% ^15^N Macklin, Shanghai, China) (Lu et al. 2020). To effectively hold the small dish in place, 16 ml of MEA was added to the fungal compartment and a 5 mm diameter mycelial plug was placed in the microcosm where the fungus grew (the control was mock-inoculated with a sterile PDA plug). Petri dishes were placed inside a square Petri dish with a 240 mm-side. A sterile moistened cotton-wool ball was placed inside the Petri dish to prevent wilting. The entire system was sealed with a double layer of Parafilm and the roots were covered with aluminium foil. Microcosms were maintained in a growth chamber for 2 weeks at 25 °C under a 14 h 12,000 lx light/10 h dark cycle. Given that shoot and leaf tissues vary in isotopic composition, the aboveground parts of the plant were divided into shoots and leaves to ensure comparability. Mycelia were also collected from fungal compartments. All the samples were dried at 65 °C for 48 h and ground into a fine powder.

Samples of ^15^N and ^13^C were measured using an IsoPrime100 isotope ratio mass spectrometer (Isoprime Ltd., Cheadle Hulme, UK) following the manufacturer’s instructions. The δ^15^N and δ^13^C values were calculated using the following equation:

δ^15^N or δ^13^C (‰) = (R_sample_ / R_standard_ – 1) × 1000

where R is the ratio of ^15^N/^14^N or ^13^C/^12^C of the sample and the standard.

### ﻿Pangenomes of *F.pseugraminearum* and recombination analysis

Prior to our research, seven genome assemblies of *F.pseudograminearum* were publicly available in databases (Suppl. material 1: table S2; Gardiner et al. 2018). The genome of the Fp8 isolate stored in our laboratory was sequenced using an Illumina platform. The genome assemblies and annotations of the nine *F.pseudograminearum* isolates are shown in Suppl. material 1: table S2. The construction of nine *F.pseudograminearum* pangenomes was described by Yuan et al. (2021). Curves describing pangenome size and the mean number of core, dispensable and new genes were fitted in R using the’ nls’ function (non-linear least squares). The curve was visualised using ggplot2 software. To further investigate the evolutionary history and potential recombination within *F.pseudograminearum* individuals, we adopted a network representation, allowing the integration of different conflicting phylogenies. We applied the NeighborNet method (Bryant and Moulton 2004) implemented in SplitsTree v. 4.13.1 (Huson 1998) on the matrix of pairwise genetic distances, calculated from the concatenated alignment of the 5,757 orthologs.

### ﻿Comparative phylogenomics

We used eight closely-related *Fusarium* species and ten isolates to construct the phylogeny. Species phylogeny was estimated using 5,974 single-copy orthologs by the Maximum Likelihood (ML) method using PhyML v.3.1, with the default model HKY85. Genes and gene clusters involved in secondary metabolism were predicted using antiSMASH v.4.0.2. The dbCAN2 (http://cys.bios.niu.edu/dbCAN2) tool was used to annotate the CAZyme repertoire. We further annotated several specific gene categories of nine *F.pseudograminearum*, including small secreted effectors, cytochrome P450s and GPCRs (Suppl. material 1: table S3), which are putatively involved in fungal virulence. Specifically, SECRETOOL was used to predict secreted proteins (http://genomics.cicbiogune.es/SECRETOOL/) and small cysteine-rich proteins (SSCPs) with fewer than 200 amino acids and more than 4% cysteine were considered small secreted effectors. The cytochrome P450s were identified using an online tool (http://drnelson.utmem.edu/CytochromeP450.html). G-protein-coupled receptors (GPCRs) were evaluated to verify the presence of seven transmembrane helices using TMHMM v.2.0, Phobius (http://www.cbs.dtu.dk/services/TMHMM/ and http://phobius.sbc.su.se/).

### ﻿Genomic structural variations between eFp and pathogenic *F.pseudograminearum* CS3096

Single polymorphisms (SNPs) and indels between the two genomes were extracted from whole-genome alignments using nucmer in the MUMmer v.3.9.4 package. We further calculated the SNP density in 20 kb non-overlapping windows across four chromosomes. Additionally, potentially large genomic re-arrangements between the two assemblies were identified using SyRI v.1.6.

### ﻿Semi-quantitative measurements of H_2_O_2_ production in roots colonised by eFp and Fp8

For *in situ* detection of root H_2_O_2_ after exposure to the two *F.pseudograminearum* isolates, the colonised and non-colonised roots were immersed in 1 mg ml^-1^ 3, 3’-diaminobenzidine (DAB, Sigma-Aldrich) at seven time points at 30 h intervals from 30 h to 210 h, then washed and bleached with acetic acid-glycerol-ethanol (1:1:3) (v/v/v) (Fester and Hause 2005). The intensity of DAB staining was quantified using ImageJ software.

### ﻿*Sweet1* gene expression pattern in populus roots when interacting with eFp and Fp8

We conducted a time course transcriptomic experiment to measure the expression pattern of *PtSweet1*, a mycorrhiza-inducible gene. Total RNA was extracted from the *P.tomentosa*-colonised and non-colonised roots using the RNAprep Pure Plant Plus kit (TIANGEN, Beijing) according to the manufacturer’s instructions. RNA integrity was confirmed by 1% agarose gel electrophoresis and RNA content was quantified using NanoDrop ND-2000 (NanoDrop, Wilmington, DE, USA). Aliquots containing 150 ng of total RNA were used for first-strand cDNA synthesis in a total volume of 20 μl, containing 1 μl oligo (dT) (Aidlab, Beijing), 1 μl gDNA Remover and 4 μl of 5×TRUE Reaction Mix, according to the manufacturer’s instructions. After synthesis, aliquots were stored at -80 °C.

*PtSweet1*-specific primers (forward 5’-AACAAGTCTCTATTTCTTTGTAACA-3’ and reverse 5’-CCATACCAAGCAGAAAGGA-3’), described by Zhang et al. (2016), were used in this study. The Actin (forward 5’-ACCCTCCAATCCAGACACTG-3’ and reverse 5’-TTGCTGACCGTATGAGCAAG-3’) (Zhang et al. 2016) and EF-1α (forward 5’-AGGTCCACCAACCTTGACTG-3’ and reverse 5’-AGGTCCACCAACCTTGACTG-3’) (Li et al. 2024) were used as reference genes to calibrate the expression of *PtSweet1*. Quantitative PCR was performed using 10 μl Q-PCR Master mix (Thermo Fisher Scientific), 8.5 μl cDNA, 0.5 μl ROXII and 10 pmol of each primer in a Light Cycler 480 System (Roche, Basel, Switzerland). Real-time PCR experiments were performed with at least four independent RNA samples and the threshold cycle (CT) was determined in triplicate. The relative levels of transcription were calculated by using the 2^−∆∆Ct^ method (Livak and Schmittgen 2001). Negative controls without cDNA were used for all PCR reactions.

### ﻿*In planta* – and *in vitro* fungal gene expressions related to toxin biosynthesis

To further determine whether the potential virulence of eFp was attenuated during symbiotic interactions with poplars, we investigated the transcriptional changes of gene clusters involved in the biosynthesis of deoxynivalenol (DON) and zearalenone (ZEN) using RNA-seq data. The identification of these two gene clusters has been previously described (Brown et al. 2004; Nahle et al. 2021). Two fungal growth conditions were established, including *in vitro* culturing on PDA plates and a symbiotic status *in planta* (40 days after inoculation, as described above). For the *in vitro* assay, fungal colonies were grown on PDA medium covered with cellophane membranes for 2 weeks at 24 °C. The mycelium grown on each plate was gently scraped from the cellophane, collected, snap-frozen in liquid nitrogen and stored at -80 °C until further analysis. For the *in planta* assay, fungal transcripts were measured from the ECM-like root tips. Three independent biological replicates were used for each growth condition. Raw sequencing reads were trimmed using fastp (Chen et al. 2018) and mapped to the eFp reference genome using Hisat2 (Kim et al. 2015). Uniquely mapped and multimapped reads were assigned and counted using a custom pipeline that integrated featureCounts (Liao et al. 2014), mmquant (Zytnicki 2017) and custom Python and R scripts. Raw read counts were normalised using the TMM normalisation approach to obtain Counts Per Million reads (CPMs) and further normalised by gene CDS lengths to obtain Fragments Per Kilobase of exon per million reads (FPKM) values using DESeq2 (Love et al. 2014) and edgeR (Robinson and Oshlack 2010).

### ﻿Data analysis and statistics

Prior to statistical analyses, all phenotypic and physiological datasets were tested for normality and variance homogeneity using Shapiro-Wilk’s and Levene’s tests, respectively. All phenotypic and physiological datasets (plant phenotypes and nutrient uptake) were subjected to Student’s *t*-test using IBM SPSS Statistics 20 software programme (SPSS Inc., http://www.spss.com.cn). A significant Student’s *t*-test was performed at *P* = 0.05. All data are expressed as mean values with standard deviations (SD).

## ﻿Results

### ﻿Morphological and anatomical structures of eFp–poplar associations

**Figure 1.**
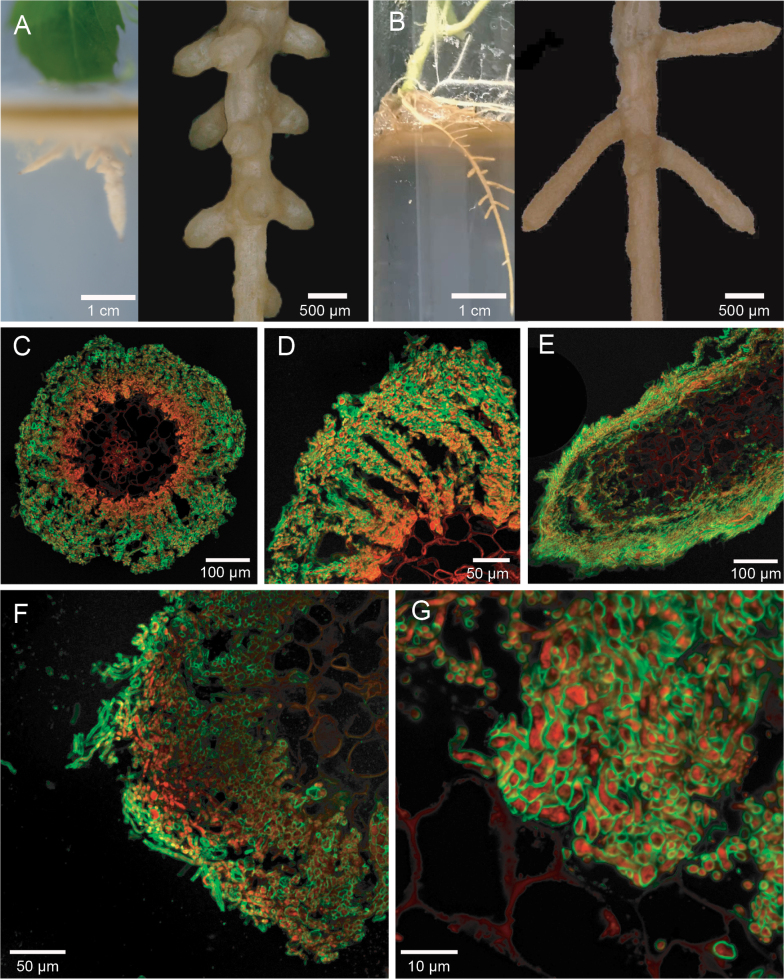
Re-synthesis experiment between eFp and *P.tomentosa*: **A, B** the ectomycorrhizal-like structure between eFp and *P.tomentosa* was photographed using a camera and an ultra-depth of field microscope 40 days after inoculation. The ECM-like root tips were single to pinnately branched, clumped and white to dark brown. The short and swollen lateral roots were ensheathed with eFp mycelia. The photos were adjusted for clarity and contrast as needed; **C, D** sectioned ECM-like root tips showing extensive hyphal colonisation along the root epidermis and cortex; **E** longitudinal section of ECM-like root tips showing a dense fungal sheath and fungal invasion of the root cap and epidermis; **F, G** a magnified view of highly-branched labyrinthine structures formed by irregular hyphal ramification in the inner cortex. All scale bars are shown in each panel.

Taking advantage of an *in vitro* system, *P.tomentosa* cuttings were inoculated by eFp under sterile conditions to determine the morphological and anatomical structures developed in this association. Three-weeks post-inoculation, short lateral roots were induced from the taproots and the mycelia often completely covered the entire lateral roots, forming root structures resembling smooth ECM root tips, which were straight with monopodial pinnate branching and becoming greyish-brown over time (Fig. 1A, B and Suppl. material 1: fig. S1). The complete development phase of this unique poplar–eFp association is shown in the Suppl. material 1: Video. The control fine roots were devoid of any apparent fungal colonisation. WGA-PI staining of cross-sections revealed sparse and thin hyphae on the root surface, resembling a patchy fungal mantle, but formation of intercellular networks of hyphae around both epidermal and cortical cells (Langer et al. 2008) (Fig. 1C–E). Colonisation of the central cylinder did not occur. More specifically, the hyphae spread across the outer and inner cortex typically formed a highly branched labyrinthine structure (Fig. 1F), which maximises the available cell surface area and optimises cell-to-cell nutrient transfer (Vaario et al. 2000). Longitudinal sections of colonised roots also showed a characteristic fungal sheath consisting of several layers of tightly packed hyphae, which further invaded the root cap and epidermal cells intercellularly (Fig. 1G).

**Figure 2.**
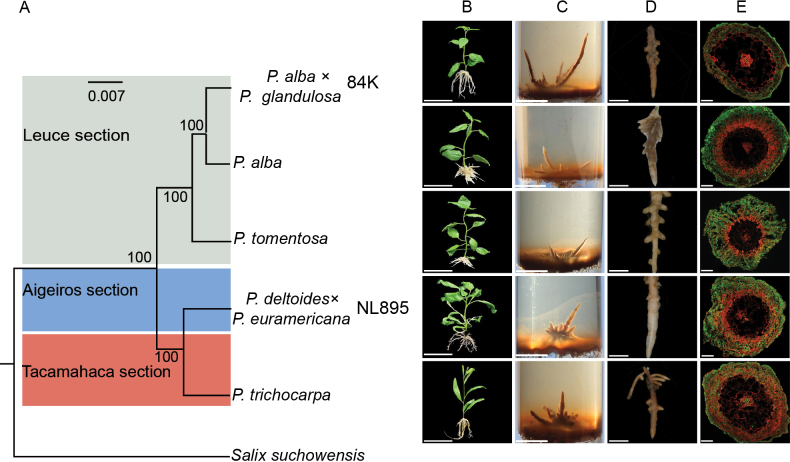
Induction of ECM-like root tips in five poplars with eFp: **A** phylogenetic relationships between five poplar trees from the three sections were constructed with 138 orthologous genes using RAxML v. 8.2.12; **B** growth status of cuttings of five poplars. The genomic data of the poplar species are presented in Suppl. material 1: table S5; **C–E** gross morphology of ECM-like root tips and a detail of cross-sections showing extensive hyphal colonisation across root epidermis and cortex in the five poplar–eFp associations. All scale bars are shown in each panel.

Subsequently, we evaluated the degree of symbiotic structure development in additional poplar species across three sections: *Leuce* (*P.alba* and *P.alba × P.glandulosa*), *Aigeiros* (*P.deltoides×P.euramericana*) and *Tacamahaca* (*P.trichocarpa*). We observed similar structural features in these associations irrespective of poplar species (Fig. 2 and Suppl. material 1: fig. S1), although the colonisation density, progression and depth of penetration into the root cortex differed amongst poplar species (Fig. 2E). For example, empirical evidence suggests that eFp resulted in the development of a symbiotic structure more rapidly with the 84k clone than with other poplar species. These findings suggest that eFp may facilitate the formation of ECM-like root tips in a non-host-specific manner.

### ﻿eFp improves growth and nutrient uptake of *P.tomentosa*

**Figure 3.**
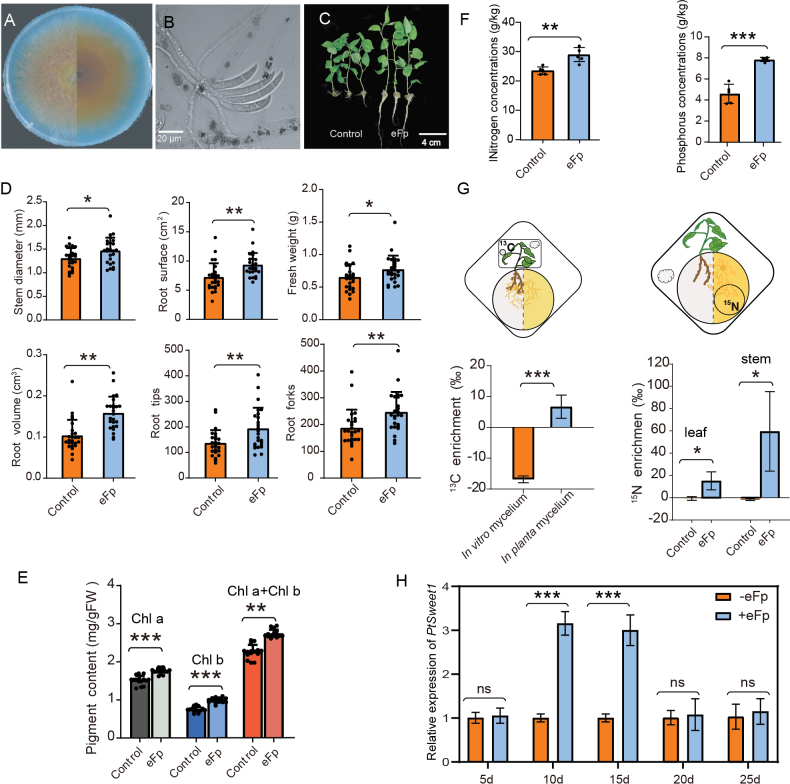
Plant growth promotion and bidirectional exchange of nutrients conferred by eFp: **A, B** colony appearance of eFp on PDA; **B** spore morphology is falcate and eFp produces only large conidia ranging from 0–6 septa; **C** comparison of the growth status between uninoculated and eFp-inoculated poplar cuttings; **D, E** statistical analyses of plant biomass, root development and chlorophyll content increased with eFp, which was analysed using Student’s *t*-test; **F** comparison of total nitrogen and phosphorus concentrations in the shoots of the control and fungus-inoculated plants; **G** Isotope tracing elements (^13^C and ^15^N) to reveal the potential bidirectional flow of nutrients between the host poplar and eFp; **H** expression pattern of the *PtSweet1* gene during symbiotic interaction with eFp.

It is evident that eFp inoculation indeed had a significant positive effect on poplar growth (Fig. 3A–C). Specifically, 40 days after inoculation, the stem diameter, biomass, total root surface, total root volume and number of root tips and forks of eFp-inoculated plants were significantly greater than those of non-inoculated cuttings, with increases of 12.53%, 18.32%, 29.28%, 52.91%, 41.95% and 31.71%, respectively (Student’s t-test, *P* < 0.05) (Fig. 3D). Moreover, the chlorophyll content of the leaves of the inoculated plants was higher than that of the control plants (*P* < 0.05) (Fig. 3E), indicating enhanced photosynthetic efficiency upon eFp colonisation. Regarding nutrition acquisition status, the total N and P concentrations in shoots from inoculated plants were significantly higher than those from control plants (*P* < 0.01 and *P* < 0.001, respectively) (Fig. 3F). These data suggest that eFp is effective in enhancing plant N and P uptake.

### ﻿Two-way flow of nutrients inferred from ^15^N and ^13^C isotopic tracing

The bidirectional nature of nutrient exchange has been recognised as a proxy for mutualistic interactions. Therefore, we quantified the transfer of ^15^N and ^13^C labelled compounds and evaluated the marker content by calculating δ^15^N and δ^13^C for stems, leaves and mycelia (Akroume et al. 2019). In our experimental microcosm, the mycelium traversed the barrier, whereas the roots were unable to penetrate the fungal compartment. Consequently, the uptake of ^15^N in poplar roots was entirely contingent upon its transport via fungal mycelia. The results demonstrated that only cuttings inoculated with eFp incorporated ^15^N in both stems and leaves (δ^15^N decreased progressively from the stem to the leaf), indicating that ^15^N can be translocated from the fungus to the aboveground plant organs. As anticipated, isotopic signatures were not detected in the control plants, thereby excluding the possibility of ^15^N leakage from the small Petri dish containing the isotope.

As illustrated in Fig. 3G, concurrent experiments demonstrated ^13^C enrichment from ^13^C-labelled photo-assimilates in the mycelium. The detection of ^13^C in the fungal compartment suggests that plants transfer carbon compounds to their roots through the assimilation of carbon dioxide. Collectively, these findings indicate that eFp and poplar trees are capable of establishing bidirectional nutrient flow.

### ﻿Expression patterns of the poplar *PtSweet1* during interactions with eFp and Fp8

The plant sugar transporter gene *Sweet1* is used as a molecular marker for characterising ECM establishment because its expression is often upregulated during symbiotic interactions (Neb et al. 2017; Li et al. 2024). Similar to mycorrhizal symbioses, the *PtSweet1* transcript level exhibited significant upregulation at 10 and 15 d post-inoculation compared to non-colonised roots (Fig. 3H). The expression pattern of *PtSweet1* in poplar 84k demonstrated a similar trend during symbiotic interactions with eFp (Suppl. material 1: fig. S2). In contrast, during the Fp8–poplar pathogenic interaction, *PtSweet1* expression increased rapidly at 5 d post-inoculation and subsequently decreased markedly at later time points (Suppl. material 1: fig. S2). This observation aligns with findings from plant–necrotrophic fungal interactions (Breia et al. 2021).

### ﻿eFp benefits a wide range of plant lineages

**Figure 4.**
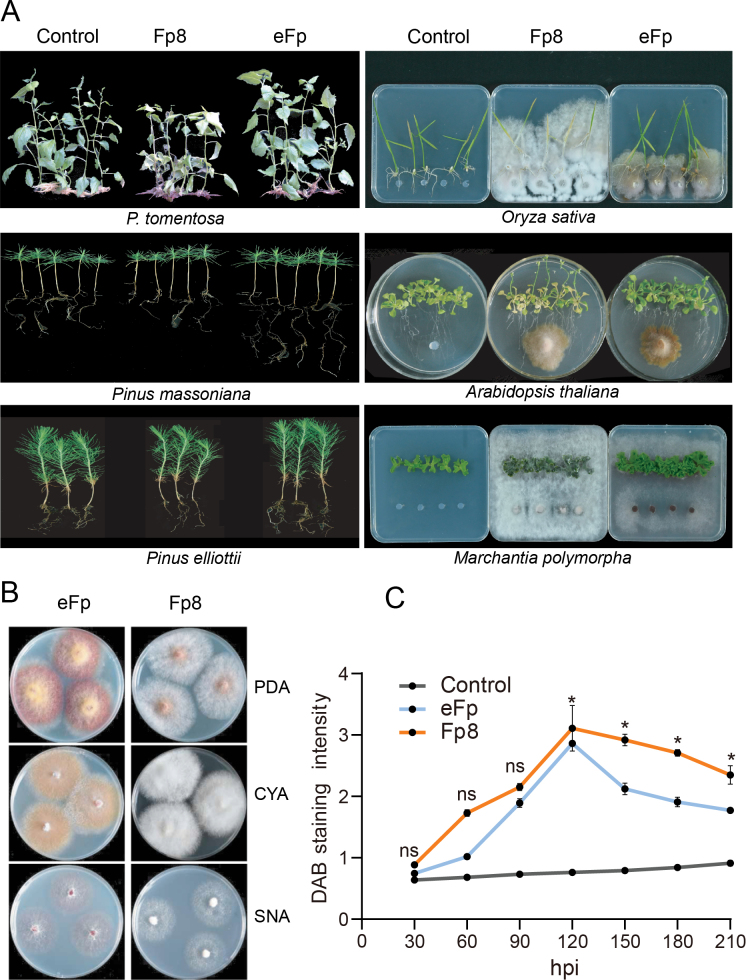
Comparison of interaction outcomes of eFp and pathogenic Fp8 with vascular and non-vascular plants: **A** for plant–eFp interactions, a stable mutualism was recorded across a wide range of plant lineages, indicating an almost complete loss of virulence of eFp. In contrast, Fp8 is detrimental to plant growth and can even cause disease symptoms in some plants; **B** macroscopic and microscopic characteristics of eFp and Fp8 on different culture media (PDA, CYA and SNA), incubated at 25 °C for 7 days; **C** root H_2_O_2_ production at seven time points in poplar roots inoculated with eFp and Fp8, as determined by DAB staining. Hpi, hour post-inoculation. Error bars represent SDs of the replicates. Data were analysed using one-way analysis of variance (ANOVA). Statistical significance was set at *P* < 0.05.

To evaluate the plant growth-promoting potential of eFp, we examined additional plant lineages, including *Pinusmassoniana* and *P.effiottii*, rice (*Oryzasativa*) and *Arabidopsisthaliana*, as well as the non-vascular liverwort *M.polymorpha* (Fig. 4A). These findings indicated that eFp enhanced the growth of all tested plants without exhibiting any pathogenic symptoms, suggesting a possible complete loss of virulence. In contrast, inoculation with strain Fp8 consistently yielded negative effects on plant growth, with varying degrees of disease symptoms, which were more pronounced in non-woody plants.

### ﻿Difference in poplar root H_2_O_2_ production during interactions with eFp and Fp8

We subsequently measured *in situ* H_2_O_2_ production as an indicator of plant response to fungal invasion during a 210 h post-inoculation time course (Fig. 4B, C). The production of H_2_O_2_ is a characteristic feature of the successful recognition of infection and activation of plant defence responses (Torres and Dangl 2005). The roots generate H_2_O_2_ during colonisation by both symbiotic and pathogenic fungi. Both colonised roots exhibited transient H_2_O_2_ elevation, with the peak occurring at 120 h post-inoculation. H_2_O_2_ levels in the roots of Fp8-infected plants were higher than those in the eFp-infected plants across all time points, with significant differences observed at the four time points (*P* < 0.05). This suggests that eFp infection appears to be considerably less effective in triggering plant defence reactions.

### ﻿eFp retains a genome structurally similar, but is highly divergent to pathogenic *F.pseudograminearum*

**Figure 5.**
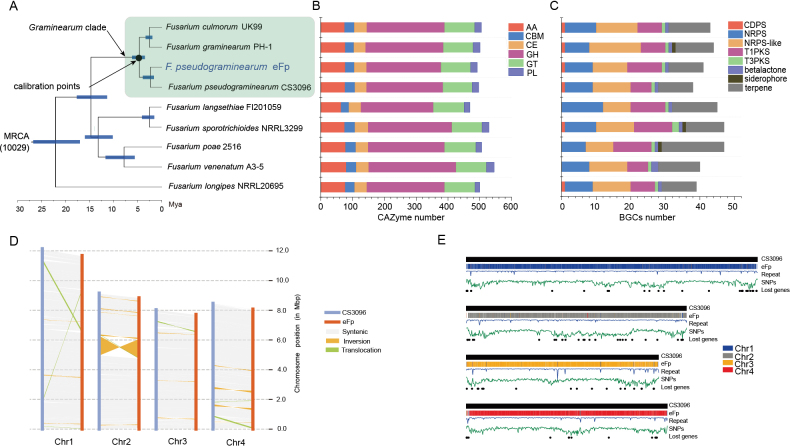
Comparison of genomic differences between eFp and pathogenic *Fusarium* spp.: **A** time-calibrated phylogeny of nine representative pathogenic *Fusarium* species, including the outgroup *Neonectriaditissima*; **B, C** comparison of the number of carbohydrate-activating enzyme genes and the number of clusters of secondary metabolite genes in the genomes of eFp and the eight pathogenic *Fusarium* species; **D** intra-species macro-synteny and re-arrangement plot depicting chromosome comparison between CS3096 and eFp using SyRI software. Syntenic blocks are indicated in grey. CS3096 chromosomes are shown in blue and eFp chromosomes are shown in orange. The four chromosomes were proportional to size; **E** whole-genome comparison of CS3096 and eFp. The black histogram bars represent the reference gene sequences of the four chromosomes of CS3096; the blue, orange, grey and red histogram bars represent the linear relationship between the gene sequences of the four chromosomes of eFp; the blue broken line (repeat) and the green folded line (SNP) represent the length proportion of the repeated sequences and the single-nucleotide polymorphisms in the reference genome CS3096 with a window of 20 kb, respectively; and the black dots (lost genes) are the missing genes relative to CS3096. SNPs located in the sub-telomeric regions of chromosomes were biased toward the ends of the chromosomes.

To identify the potential genomic traits specific to eFp, we generated a chromosome-level genome assembly. Phylogenetic analysis confirmed the placement of eFp in the *Graminearum* clade and showed that eFp is conspecific to the reference isolate *F.pseudograminearum* CS3096 (Fig. 5A; Gardiner et al. 2018). Three key findings emerged from comparative and population genomic analyses. First, the eFp genome did not exhibit significant streamlining compared with that of CS3096 and other genetically proximate taxa. Upon further comparison of a set of specific gene categories related to lifestyle and virulence, no apparent gene contraction or reduction in eFp expression was observed (Fig. 5B, C and Suppl. material 1: table S3). One exception was the slight decrease in the number of small secreted effectors. Despite these conserved trait-associated gene families, transcriptomic responses may differ significantly, as exemplified by the expression of two major toxins, deoxynivalenol (DON) and zearalenone (ZEN) in eFp, which could maintain a minimal level during the symbiotic interaction (Suppl. material 1: fig. S3). Second, the degree of synteny between the eFp and CS3096 genomes was very high, with 11,485 syntenic gene pairs identified, except for a region on chromosome 2 where a re-arrangement (a large-scale inversion comprising approximately ~ 1.33 Mb covering > 14.5% of chromosome 2) was found and the number of genes in this inverted synteny block contained a total of 500 genes (Fig. 5D).

**Figure 6.**
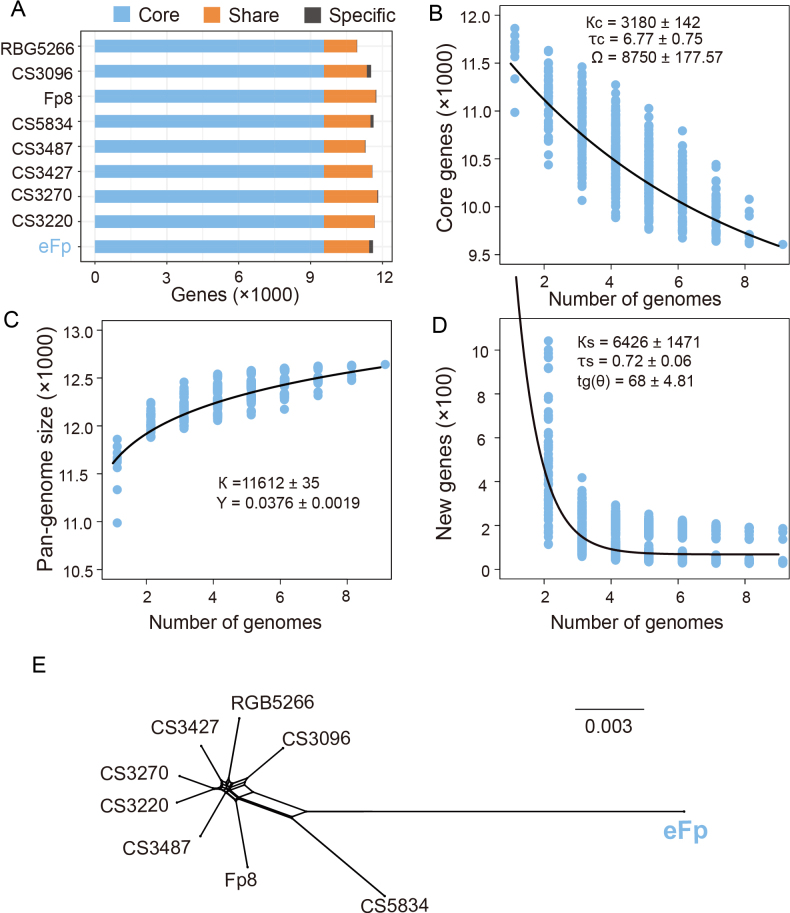
Pan-genome analysis of *F.pseudograminearum*: **A** proportion of genes in the core, dispensable and private genomes; **B** estimation of *F.pseudograminearum* core genome. Parameter κc is the amplitude of the exponential decay, τc is the decay constant and Ω is the best-fit value of the core genome; **C** estimation of the pan-genome size of *F.pseudograminearum*. The curve is a least-squares fit of the power law to the median; **D** estimation of new genes: The number of individual-specific genes is plotted as a function of the number of genomes sequentially added. Parameters κs and τs are equivalent to κc and τc, respectively and tg(θ) measures the best-fit number of specific genes; **E** a SplitsTree network showing relationships of all *F.pseudograminearum* isolates with no outgroup, which was estimated using the NeighborNet method using pairwise genetic distances (F84 distances obtained from the concatenated 5,757 orthologs aligned in codons).

Despite its high synteny, we identified an extensive number of single-nucleotide polymorphisms (SNPs) and insertions/deletions (indels) when mapping CS3096 (Fig. 5E and Suppl. material 1: table S4), indicating potential evolutionary innovations. A total of 97 genes were found to be absent and 150 genes were specific to eFp, based on pan-genome analysis of nine *F.pseudograminearum* individuals (Fig. 6A). However, no Gene Ontology (GO) terms were found to be significantly enriched. The *F.pseudograminearum* pan-genome was determined to be open, as evidenced by an estimated exponent γ > 0 (Fig. 6B–D). Furthermore, 68 novel genes were identified in each *F.pseudograminearum* genome. Additionally, reticulate (i.e. not tree-like) evolutionary relationships within the pathogenic group were demonstrated, suggesting a clear signal of recombination (Fig. 6E). This analysis also confirmed low divergence amongst pathogenic isolates, as evidenced by the short branch lengths within the network. In contrast, eFp exhibited a long interior branch that did not cluster with the other eight pathogenic individuals (Fig. 6E), confirming that the eFp followed a distinct evolutionary trajectory.

## ﻿Discussion and conclusions

### ﻿ECM-like associations can arise without prior co-evolution

In the present study, we characterised the mutualistic association established between eFp and poplar trees. Comparative genomics of hundreds of ECM fungi have shown that they share common genomic traits, such as reduced number of plant cell wall-degrading enzymes and secondary metabolism (SM) clusters and the proliferation of transposable elements, common landmarks of the switch to symbiotic lifestyle in fungi (Kohler et al. 2015; Hess et al. 2018; Miyauchi et al. 2020; Lebreton et al. 2021). However, the evolution of these genomic traits along the saprotrophy-to-symbiotrophy continuum remains unclear. Our comparative genomic analyses suggest that, despite significant divergence from pathogenic *F.pseudograminearum*, no single feature distinguishes the eFp genome from the genomes of pathogenic strains. We did not observe erosion of genome size or gene repertoire size between pathogenic *F.pseudograminearum* and eFp individuals. Similarly, the repertoire of SM clusters and CAZyme genes was similar amongst *F.pseudograminearum* strains and only a slight decrease in the number of secreted effectors was observed in eFp. This finding implies that eFp shares a limited set of genomic traits with typical ECM fungi. These findings support previous studies demonstrating that CAZymes and effector contents are not highly relevant for exploring genetic differences in plant-associated lifestyles in fusarioid fungi (Hill et al. 2022). Moreover, attenuation of virulence is a prerequisite for endophytic development. Although pathogenic traits were retained in the present study, such as a complete set of toxin biosynthetic clusters in the eFp genome, their expression was significantly reduced during ECM development, rendering eFp less detrimental to its hosts (Nunes-Alves 2015; Lofgren et al. 2018; Genre et al. 2020; Redkar et al. 2022b).

Our findings suggest that mycorrhizal plants can respond to non-adapted microbes, resulting in mutualistic endophytic associations without prior co-evolution. A similar pattern was observed in the establishment of mutualism between the alga *Chlamydomonasreinhardtii*-engineered filamentous fungi (Aanen and Bisseling 2014). More specifically, the established symbiosis might not require intimate co-evolution of the host and microbes, but rather might be the result of the root perception of conserved microbial signals (Nod and Myc factors), which are able to activate the symbiosis-specific host signalling pathway, known as the common symbiotic signalling pathway (Skiada et al. 2020). It is reasonable to assume that eFp could induce similar symbiotic responses in ectomycorrhizal plants. Of note, at this time, the eFp–poplar associations have only been re-synthesised under laboratory conditions. Unfortunately, the ECM-like structure was absent in greenhouse and field experiments, indicating that this specialised symbiosis requires specific nutritional requirements.

### ﻿eFp serves as a beneficial mycobiont

Most *Fusarium* species are plant or human pathogens (Summerell 2019). However, emerging evidence suggests that endophytic and soil non-pathogenic *Fusarium* species are also ubiquitous (Maciá-Vicente et al. 2008), indicating their multifaceted lifestyles. The *F.oxysporum* (Fo) complex serves as a compelling model for understanding pathogenic and non-pathogenic relationships (Hom and Murray 2014; Brader et al. 2017) and it has become evident that not all Fo are pathogenic (Wang et al. 2020; Guo et al. 2021). For example, Fo47 exhibits both biocontrol and plant growth-promoting capabilities. This is not always the case, however, as such mutualistic interactions may fail. Redkar et al. (2022b) observed that Fo47 is detrimental to the non-vascular liverwort *M.polymorpha*, suggesting the retention of a set of core pathogenicity factors. In contrast, eFp confers benefits to a wide range of plants, reflecting near-complete loss of virulence. The principle of reciprocal nutrient exchange (Almario et al. 2022) is applicable to eFp–poplar association and eFp functions as a robust enhancer of plant growth. Overall, our data appear to support the notion that eFp has already entered a transitional state and could readily evolve into mutualistic endophytic associations and possibly into canonical mycorrhizal associations (Selosse et al. 2018, 2022; Wu and Cox 2021).

An extensive number of structural genomic variants, including deletions and point mutations, in combination with re-arrangements can greatly affect gene function and even create pseudogenes. These factors could have contributed to the uniqueness of the eFp genome structure. Likewise, some *Colletotrichum* species can also express both pathogenic and mutualistic interactions with plants resulting from subtle genetic variations (Hacquard et al. 2016), which facilitates a shift in lifestyle from harmful pathogens to mutualistic fungi (Hiruma et al. 2023). Therefore, it is suggested that the cryptic lifestyle of *Fusarium* remains largely unexplored.

### ﻿Endophyte–ECM plant interactions versus ECM fungus–non-ECM plant interactions

There has been an impressive amount of work on both endophytic and ECM associations. Model herbaceous plants and crops have been widely used as materials for endophytic fungal inoculation. By comparison, the interactions between ECM plants and endophytic fungi and those taking place between non-ECM plants and ECM fungi are rarely addressed. In the former system, there are increasing studies on poplar root-associated fungal endophytes and their symbiotic performance (Hacquard and Schadt 2015; Vélez et al. 2017). However, the anatomical structures of these associations have not been adequately investigated (Lacercat-Didier et al. 2016; Liao et al. 2019). To the best of our knowledge, this is the first report to show the establishment of fungal structures in root tips resembling those of ECM symbiosis by an endophytic *Fusarium*. In the latter system, it has been shown that some ECM fungi, such as *Tuber* species, colonise the roots of non-ECM herbaceous plants, form stable endophytic structures (Schneider-Maunoury et al. 2020) and improve the development of lateral roots (*Pisolithustinctorius*–*Arabidopsisthaliana*) (Reboutier et al. 2002). These types of associations are relevant for investigating evolutionary transitions.

To create a broader catalogue of potential mycorrhizal fungi, recently, Kariman et al. (2024) advocated the necessity of expanding mycorrhizal boundaries because some endophytes possess core features of mycorrhizal symbioses. Some endophytes have isotopic patterns that resemble ECM fungi (Vanegas-León et al. 2019). One of the most compelling examples is the dark septate endophytic fungus *Acephalamacrosclerotiorum*, which establishes ECM associations (Lukešová et al. 2015). In parallel, the term ‘brassicoid mycorrhizas’ has been coined by Almario et al. (2022) to describe the association formed between *Arabidopsisthaliana* and the endophytic fungus *Colletotrichumtofieldiae* strain Ct61 (Hiruma et al. 2016). Moreover, a list of *Fusarium* species has been suggested as candidate mycorrhizal fungi in orchids (Jiang et al. 2019; Sisti et al. 2019). Thus, *C.tofieldiae* Ct61 and eFp may fit the category of mycorrhizal-like endophytes and the plant–fungus symbioses initiated by them support the ‘waiting room hypothesis’ (Selosse et al. 2022). Thus, a special focus on endophyte–ECM plant interactions is crucial to obtain structural and functional evidence of the mycorrhizal or endophytic status of root-associated fungi from ECM plants.

In conclusion, our work points out a novel type of specialised symbiosis developed in eFp–poplar associations that may mimic ectomycorrhizas, suggesting that eFp has undergone sufficient divergence and exhibits distinct symbiotic characteristics in its genome, although a plausible mechanism has not yet been fully elucidated. Further investigation into this unique form of mutualism will be facilitated through robust genetic manipulation of *Fusarium*. Further research carried out on this symbiotic model will yield more insights into the evolutionary transitions from endophytic to mycorrhizal associations, more broadly, understanding fungal guilds with potential dual ECM/endophyte niches.

## ﻿Additional information

### Author ORCIDs

Ningning Yang https://orcid.org/0009-0006-3229-1748

Xiaoliang Shan https://orcid.org/0000-0002-7553-8046

Kexuan Wang https://orcid.org/0000-0002-8512-2175

Francis M. Martin https://orcid.org/0000-0002-4737-3715

Zhilin Yuan https://orcid.org/0000-0002-5799-8401

### Data availability

The RNA-seq reads from the six libraries were deposited in SRA with accession numbers SRR23815950–SRR23815955. The accession numbers for the assembled genome of eFp are CP064753–CP064759. The accession number for the assembled genome of Fp8 is SRR29263794. All data have been submitted under BioProject accession number PRJNA643593. The Source data (Suppl. material 2) and Suppl. material 1 are in the DRYAD repository (https://doi.org/10.5061/dryad.rjdfn2znz).

## Supplementary materials

Supplementary material 1 Supplementary figures, tables and video Data type zip https://binary.pensoft.net/file/1273067This dataset is made available under the Open Database License (http://opendatacommons.org/licenses/odbl/1.0/). The Open Database License (ODbL) is a license agreement intended to allow users to freely share, modify, and use this Dataset while maintaining this same freedom for others, provided that the original source and author(s) are credited.Ningning Yang, Xiaoliang Shan, Kexuan Wang, Junkun Lu, Ying Zhu, Redman S. Regina, Russell J. Rodriguez, Jiajia Yao, Francis M. Martin, Zhilin Yuan

Supplementary material 2 Source data Data type xlsx https://binary.pensoft.net/file/1273068This dataset is made available under the Open Database License (http://opendatacommons.org/licenses/odbl/1.0/). The Open Database License (ODbL) is a license agreement intended to allow users to freely share, modify, and use this Dataset while maintaining this same freedom for others, provided that the original source and author(s) are credited.Ningning Yang, Xiaoliang Shan, Kexuan Wang, Junkun Lu, Ying Zhu, Redman S. Regina, Russell J. Rodriguez, Jiajia Yao, Francis M. Martin, Zhilin Yuan
