# Supplementary material for: ﻿A fusarioid fungus forms mutualistic interactions with poplar trees that resemble ectomycorrhizal symbiosis
Source: IMA Fungus. 2025 Mar 7;16:e143240. doi: 10.3897/imafungus.16.143240 (PMC11909594; doi:10.3897/imafungus.16.143240)
Supplement: Supplementary material 1 — Supplementary figures, tables and video [file imafungus-16-e143240-s001.zip › Supplementary Information/Fig. S1 original images of ECM-like organisms under the ultra-deep-field microscope.pdf]

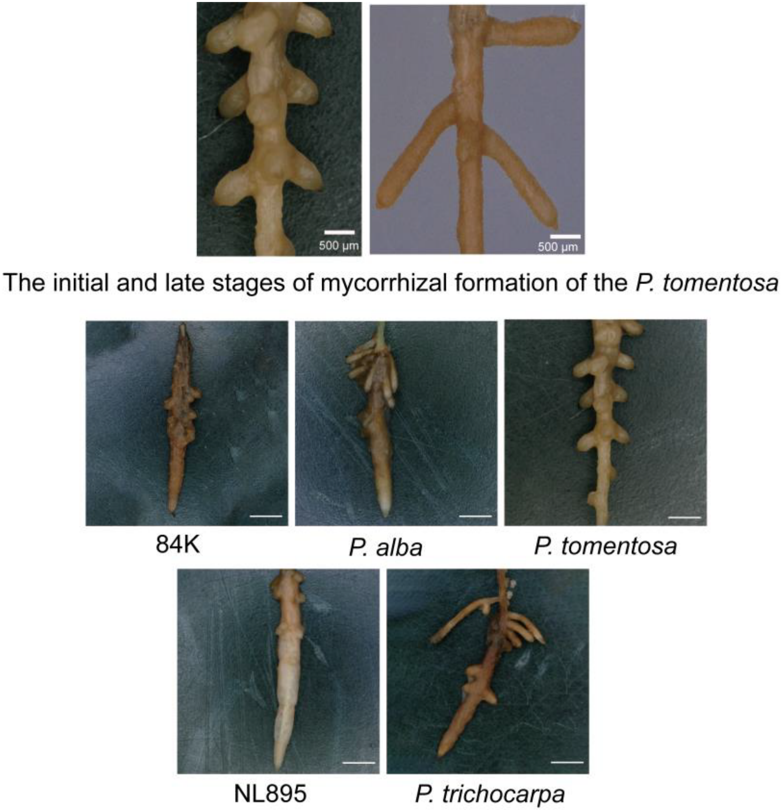

Fig. S1 The original images of the ectomycorrhizal-like structure between eFp and five poplars were photographed using in an ultra-depth of field microscope 40 days.
