# Supplementary material for: ﻿A fusarioid fungus forms mutualistic interactions with poplar trees that resemble ectomycorrhizal symbiosis
Source: IMA Fungus. 2025 Mar 7;16:e143240. doi: 10.3897/imafungus.16.143240 (PMC11909594; doi:10.3897/imafungus.16.143240)
Supplement: Supplementary material 1 — Supplementary figures, tables and video [file imafungus-16-e143240-s001.zip › Supplementary Information/Table S1 The media.docx]

**Table S1** The media compositions for micropropagation of five poplar clones.

| Species name | Media compositions (1L) |
| --- | --- |
| *P. tomentosa* | 1/2 MS 2.47 g+IBA 0.5 mg  +Sucrose 15g |
| *P. alba×P. glandulosa*  84k | 1/2 MS 2.21g+IBA 0.05mg  +NAA 0.02mg |
| *P. alba* | 1/2 MS 2.47 g+IBA 0.5 mg  +Sucrose 15g |
| *P. deltoides×P. euramericana* NL895 | Woody Plant Medium (WPM) with vitamins 1.991g+Ca^2+^salt 1ml+MES monohydrate 0.5g+ Sucrose 20g |
| *P. trichocarpa* | WPM 2.41g+IBA 0.1mg+ Sucrose 15g |
