# Supplementary material for: ﻿A fusarioid fungus forms mutualistic interactions with poplar trees that resemble ectomycorrhizal symbiosis
Source: IMA Fungus. 2025 Mar 7;16:e143240. doi: 10.3897/imafungus.16.143240 (PMC11909594; doi:10.3897/imafungus.16.143240)
Supplement: Supplementary material 1 — Supplementary figures, tables and video [file imafungus-16-e143240-s001.zip › Supplementary Information/Table S2 Summary of genomic.docx]

| Individuals | Genome size (Mb) | Chromosomes or scaffolds | TE length (Mb) | Gene number | Complete BUSCOs (%) |
| --- | --- | --- | --- | --- | --- |
| eFp | 37.94 | 4 Chromosomes+3 scaffolds | 1.75 | 12,108 | 99.8 |
| CS3220 | 37.26 | 191 Scaffolds | 1.33 | 12,090 | 99.0 |
| CS3270 | 37.07 | 4 Chromosomes | 1.05 | 12,238 | 99.8 |
| CS3427 | 37.13 | 182 Scaffolds | 1.24 | 11,948 | 97.5 |
| CS3487 | 37.11 | 364 Scaffolds | 1.25 | 11,632 | 94.8 |
| CS5834 | 37.58 | 228 Scaffolds | 1.41 | 12,084 | 98.9 |
| FP8 | 37.33 | 4122 Scaffolds | 0.98 | 11,989 | 99.6 |
| CS3096 | 36.97 | 4 Chromosomes+275 scaffold | 0.822 | 12,397 | 98.9 |
| RBG | 36.34 | 4 Chromosomes+73 scaffold | 0.44 | 11,483 | 99.4 |

**Table S2** Summary of genomic assembly and annotation results of nine *F. pseudograminearum* individuals

Note: the benchmarking universal single-copy orthologs (BUSCO v 5.beta.1) tool with the Fungi odb10 data set was used to evaluate the integrity of the genome assembly.
