# Supplementary material for: ﻿A fusarioid fungus forms mutualistic interactions with poplar trees that resemble ectomycorrhizal symbiosis
Source: IMA Fungus. 2025 Mar 7;16:e143240. doi: 10.3897/imafungus.16.143240 (PMC11909594; doi:10.3897/imafungus.16.143240)
Supplement: Supplementary material 1 — Supplementary figures, tables and video [file imafungus-16-e143240-s001.zip › Supplementary Information/Table S3 Summary of protein.docx]

**Table S3** Comparison of four protein-family numbers identified in nine *F. pseudograminearum* individuals.

| Individuals | Secreted proteins | SSCPs | Cytochrome P450 | GPCRs |
| --- | --- | --- | --- | --- |
| CS3220 | 489 | 91 | 368 | 225 |
| CS3270 | 499 | 88 | 374 | 232 |
| CS3427 | 490 | 88 | 359 | 221 |
| RBG5266 | 474 | 78 | 351 | 201 |
| CS3487 | 473 | 85 | 356 | 213 |
| CS3096 | 496 | 89 | 361 | 211 |
| Fp8 | 495 | 88 | 366 | 216 |
| CS5834 | 468 | 86 | 365 | 225 |
| eFp | 476 | 79 | 368 | 224 |
