# Supplementary material for: ﻿A fusarioid fungus forms mutualistic interactions with poplar trees that resemble ectomycorrhizal symbiosis
Source: IMA Fungus. 2025 Mar 7;16:e143240. doi: 10.3897/imafungus.16.143240 (PMC11909594; doi:10.3897/imafungus.16.143240)
Supplement: Supplementary material 1 — Supplementary figures, tables and video [file imafungus-16-e143240-s001.zip › Supplementary Information/Table S4 Summary of SNP.docx]

**Table S4** Summary of number of identified SNPs and indels in eFp compared to CS3096

| Total SNPs | 928,309 |
| --- | --- |
| SNP in genes | 481,044 |
| SNP in CDS | 389,108 |
| Synonymous | 277,920 |
| nonsynonymous | 111,188 |
| nonsynonymous relate gene number | 10,413 |
| Total Indels | 81,582 |
| Insertion | 39,407 |
| Insertion in genes | 6,945 |
| Insertion in CDS | 2,821 |
| Insertion in CDS gene number | 1,567 |
| Deletion | 42,175 |
| Deletion in gene | 7,215 |
| Deletion in CDS | 2,958 |
| Deletion in CDS gene number | 1,507 |
