# Supplementary figures and images for: ﻿A fusarioid fungus forms mutualistic interactions with poplar trees that resemble ectomycorrhizal symbiosis
Source: IMA Fungus. 2025 Mar 7;16:e143240. doi: 10.3897/imafungus.16.143240 (PMC11909594; doi:10.3897/imafungus.16.143240)

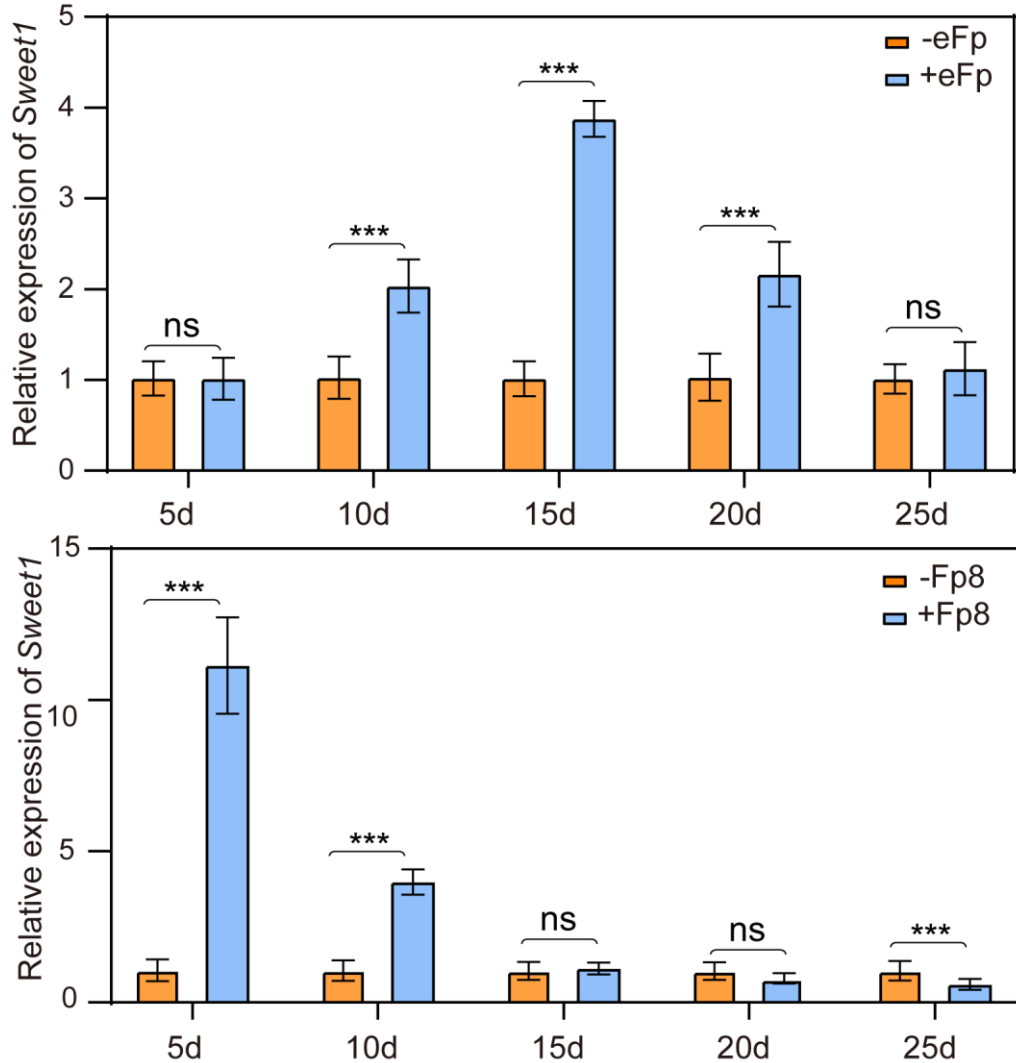

**Fig. S2** Expression patterns of the poplar 84k *PtSweet1* during interactions with eFp and Fp8

Supplement: Supplementary material 1 — Supplementary figures, tables and video [file imafungus-16-e143240-s001.zip › Supplementary Information/Fig. S2 84K SWEET1.pdf]
