# Supplementary material for: ﻿A fusarioid fungus forms mutualistic interactions with poplar trees that resemble ectomycorrhizal symbiosis
Source: IMA Fungus. 2025 Mar 7;16:e143240. doi: 10.3897/imafungus.16.143240 (PMC11909594; doi:10.3897/imafungus.16.143240)
Supplement: Supplementary material 1 — Supplementary figures, tables and video [file imafungus-16-e143240-s001.zip › Supplementary Information/Table S5 Genomic and transcriptomic data.doc]

**Table S5** Genomic and transcriptomic data for constructing the phylogenetic relationship of five poplar species

| Species name | Genomics Data Links |
| --- | --- |
| *P. tomentosa* | <https://www.ncbi.nlm.nih.gov/nuccore/JAAWWB000000000.1/>  GenBank under the accession JAAWWB000000000 |
| *P. alba×P. glandulosa*  84k | <http://ftp.cngb.org/pub/CNSA/data1/CNP0000339/CNS0047055/CNA0003521/>  https://db.cngb.org/search/assembly/CNA0003521/ |
| *P. alba* | https://www.ncbi.nlm.nih.gov/nuccore/1635325343  GenBank under the accession RCHU00000000 |
| *P.deltoides×P.euramericana* NL895 | <https://www.ncbi.nlm.nih.gov/bioproject/?term=PRJNA471819>  GenBank under the accession PRJNA471819 |
| *P. trichocarpa* | https://www.ncbi.nlm.nih.gov/Traces/wgs/AARH04  GenBank Accession Number: AARH00000000 |
| *Salix suchowensis* | <https://ftp.ncbi.nlm.nih.gov/genomes/all/GCA/017/552/425/GCA_017552425.1_ASM1755242v1/>  https://www.ncbi.nlm.nih.gov/nuccore/JAEQKX000000000.1  GenBank under the accession JAEQKX000000000 |
